# Perceived discrimination is associated with severity of positive and depression/anxiety symptoms in immigrants with psychosis: a cross-sectional study

**DOI:** 10.1186/1471-244X-11-77

**Published:** 2011-05-06

**Authors:** Akiah O Berg, Ingrid Melle, Jan Ivar Rossberg, Kristin Lie Romm, Sara Larsson, Trine V Lagerberg, Ole A Andreassen, Edvard Hauff

**Affiliations:** 1 Institute of Clinical Medicine, Faculty of Medicine, University of Oslo, Oslo, Norway; 2 Division of Mental Health and Addiction, Oslo University Hospital, Oslo, Norway

## Abstract

**Background:**

Immigration status is a significant risk factor for psychotic disorders, and a number of studies have reported more severe positive and affective symptoms among immigrant and ethnic minority groups. We investigated if perceived discrimination was associated with the severity of these symptoms among immigrants in Norway with psychotic disorders.

**Methods:**

Cross-sectional analyses of 90 immigrant patients (66% first-generation, 68% from Asia/Africa) in treatment for psychotic disorders were assessed for DSM-IV diagnoses with the Structured Clinical Interview for DSM Disorders (SCID-I, sections A-E) and for present symptom severity by The Structured Positive and Negative Syndrome Scale (SCI-PANSS). Perceived discrimination was assessed by a self-report questionnaire developed for the Immigrant Youth in Cultural Transition Study.

**Results:**

Perceived discrimination correlated with positive psychotic (r = 0.264, p < 0.05) and depression/anxiety symptoms (r = 0.282, p < 0.01), but not negative, cognitive, or excitement symptoms. Perceived discrimination also functioned as a partial mediator for symptom severity in African immigrants. Multiple linear regression analyses controlling for possible confounders revealed that perceived discrimination explained approximately 10% of the variance in positive and depression/anxiety symptoms in the statistical model.

**Conclusions:**

Among immigrants with psychotic disorders, visible minority status was associated with perceived discrimination and with more severe positive and depression/anxiety symptoms. These results suggest that context-specific stressful environmental factors influence specific symptom patterns and severity. This has important implications for preventive strategies and treatment of this vulnerable patient group.

## Background

Immigration status is a risk factor for schizophrenia, other psychotic disorders, and bipolar disorder [1,2]. Elevated risk was observed for a variety of ethnic groups and was highest for visible minorities and immigrants experiencing greater cultural barriers [3]. Two meta-analyses found highest relative risk for schizophrenia among migrants from countries where the majority are black, compared to migrants from areas where the majority are white or Asian [1,4]. Increased risk was equal for both first and second generation immigrants, and this finding has led to a growing consensus that the development of psychotic disorders in immigrants is associated with sensitization to environmental stressors related to the post-immigration context [4-8]. Perceived discrimination is an important post-immigration stressor that is associated with heightened risk for psychosis [9,10].

Minority status may result in overt discrimination and contribute to feelings of alienation from the majority culture. Discrimination is usually defined as a difference in treatment based on factors other than individual merit, including nationality or ethnicity, and may lead to the relative deprivation of resources and rewards [11]. Discrimination can be both actual and perceived, but is frequently measured only as perceived because confirming actual discrimination is difficult in a research setting. Immigrants and ethnic minorities often experience social adversity, and perceived discrimination may be an especially relevant context-dependent stressor for visible minority groups. A recent meta-analysis revealed that perceived discrimination was associated with an increased probability of clinical mental illness [12]. This is relevant to the hypothesis that social defeat, defined as a chronic experience of social exclusion or an inferior or subordinate position in society, may lead to dopaminergic hyperactivity in the mesocorticolimbic system, the same system found to be sensitised in schizophrenia [13].

A number of studies suggest a significant association between perceived discrimination and psychosis in immigrant or ethnic minority groups. Studies from the Netherlands found that the incident rate for all psychotic disorders was highest among ethnic groups that reported the most severe discrimination [10]. Studies covering different psychotic disorders at different stages of development in different immigrant groups also indicate that high rates of discrimination may be associated with the onset- and/or symptomatic features of the disorders [9,14-16].

A number of studies suggest that immigrants and ethnic minority groups with psychosis have a distinct psychopathological profile from patients of the ethnic majority. There are reports of more hallucinations, primarily auditory, among psychotic patients from a number of ethnic minority and immigrant groups both in the USA and Europe [17-25]. Perceived discrimination is also associated with the positive symptoms of delusional and paranoid ideation [16,26]. In addition, there are reports of more severe depressive symptoms among both ethnic minority and immigrant patients with psychotic disorders [18,24,27].

These studies have demonstrated that patients from ethnic minority groups appeared to exhibit more severe positive and affective symptoms across a broad range of psychotic disorders. However, the mechanisms underlying this specific symptom profile are unknown. It is possible that context-specific stressors, including perceived discrimination, may contribute to these distinct symptom profiles. Perceived discrimination may be an especially relevant context-dependent stressor for *visible *minority groups, and may partly explain why immigrant groups with dark skin colour in areas where they are a visible minority are at particular high risk [28,29].

In this study, we investigated if perceived discrimination was associated with the severity of positive and affective symptoms among immigrants diagnosed with a psychotic disorder. We surmised that among visible minorities, the severity of these specific symptoms was mediated by perceived discrimination. Furthermore, we hypothesized that perceived discrimination contributed to positive and affective symptom severity among immigrants, even in the presence of other relevant factors that may influence symptom severity.

## Methods

This study is part of the ongoing "Thematically Organized Psychosis" (TOP) Study at the University of Oslo, and is approved by the Regional Committee for Medical Research Ethics and the Norwegian Data Inspectorate. Our research methodology conformed to The Code of Ethics of the World Medical Association, Helsinki Declaration [30]. The study had a cross-sectional design including a large, non-selected and consecutive catchment area sample of patients with a DSM-IV psychotic disorder.

### Procedure

Participants were recruited consecutively from both inpatient and outpatient units at four hospitals in Oslo that collectively cover a catchment area of 485,000 people (88% of Oslo's total population). Clinicians from the recruitment units were asked to refer all patients with a clear or potential diagnosis of a psychotic disorder, and were reminded at regular intervals. These units served all patients living in the catchment areas and there were no alternative psychiatric services offering treatment for psychotic disorders. Those who agreed to participate were assessed by a trained psychologist or psychiatrist. Inclusion criteria were clear DSM-IV diagnosis of psychosis, no signs of organic etiology or substance induced symptoms, between 18-65 years of age, IQ >70, and the ability to understand and speak a Scandinavian language. All participants gave informed consent. Exclusion criteria to this study were migration by adoption and indigenous ethnic minority status (Sami people).

### Immigrant definitions

We based migration history on observed ethnicity, country of birth, mother tongue, and immigrant status of parents. First generation immigrants (FGIs) were defined as immigrants to Norway with no preceding parents or family members. Second generation immigrants (SGIs) were defined as Norwegian-born children of FGIs, or foreign-born children of one FGI and one Norwegian parent. For Norwegian-born participants with an immigrant background, we registered the parent's country of birth.

To investigate differences between immigrants' origins, we followed Statistics Norway's present division of "Europe, Africa, Asia plus Turkey, North America, and South America". We refer to these categories as geographical origins, and in this context both FGIs and SGIs from Asia and from Africa were considered immigrant groups with visible minority status in Norway.

### Instruments

Diagnoses was assessed with The Structured Clinical Interview for DSM-IV (SCID-I), affective, psychotic, and substance abuse sections (A-E) [31]. The reliability and validity of DSM-IV diagnoses across ethnic groups was ensured by the previous participation of all study clinicians in an international training program that included diagnosis of patients of different ethnic backgrounds [32]. The overall agreement for DSM-IV diagnoses was 82% with an overall kappa of 0.77 (95% CI: 0.60-0.94). Difficult differential diagnoses were decided by consensus among study clinicians. All assessments included a full life history of actual study patients and videotapes (training videos), so assessors were not blind to information about migration history.

The Structured Positive and Negative Syndrome Scale (SCI-PANSS) [33] was used to measure present symptom presentation and severity in this mixed cohort because it measures similar symptom domains in patients with schizophrenia or bipolar disorder [34]. The PANSS was originally assessed as reliable among a group of schizophrenic patients with diverse ethnicities (43% African-American, 33% European-American, 24% Hispanic-American), thus supporting this instrument's cross-ethnic reliability. To further assess symptoms, we subdivided the PANSS scale into positive, anxiety/depression, excitement, negative, and cognitive factors based on items found to be valid across different cultures [35]. Anxiety/depression and excitement factors were considered to express affective symptoms. Our study group had acceptable interclass correlation coefficients for all scales: 0.73 for positive and negative scales, 0.71 for the general scale.

Symptom severity and function were rated separately with a split version of the Global Assessment of Functioning Scale (GAF) [36]. Inter-rater reliability, as measured by the interclass coefficient, was 0.86 for GAF-symptoms (95% CI: 0.77 - 0.92) and 0.85 for GAF-function (95% CI: 0.76 - 0.92).

Assessment of perceived discrimination was based on a self-report questionnaire developed for the Immigrant Youth in Cultural Transition Study [37]. It contained five questions that assessed such issues as "feeling unjustly treated" or "insulted because of ones cultural background". Questions were constructed as a Likert scale with four possible choices from "strongly agree" to "strongly disagree". It was a forced choice scale with no middle options of "agree" or "disagree". The questionnaire was previously used in the Oslo Health Survey youth section [38]. It has been found to be a reliable instrument among adults with schizophrenia-spectrum disorder and healthy controls, and to measure the same psychological constructs in all non-western ethnic groups participating in these studies as defined by the Netherlands' Bureau of Statistics [39]. In our study, the scale showed acceptable internal consistency (Cronbach's α of 0.73). We also inquired about perception of discrimination in housing and denial of employment due to immigrant status (subsequently termed denial of resources), using two questions from the Oslo Immigrant Health study [40].

### Participants

From November 2006 to January 2010, a total of 566 participants were included in the TOP study. Of these, 25% (N = 145) had immigrant backgrounds, which is slightly higher than the percentage of immigrants in the general population of Oslo (23%). There were also slightly more immigrants from Asia plus Turkey, Africa, and South- and Latin- America in the TOP sample (18%) compared to the general population (16%). The TOP sample had approximately 5% fewer FGIs (and 5% more SGIs) than the general population [41]. The final study sample consisted of 90 immigrants who had completed the questionnaire (63% participation) and consisted of 10% fewer FGIs (and 10% more SGIs) than the general immigrant population of Oslo.

Immigrants in our sample were significantly younger than non-immigrant patients (29.7 ± 9.8 vs. 32.16 ± 11.3, t = 2.514, df = 292.635, p < 0.012), and had fewer years of education (12.54 ± 3.4 vs. 13.26 ± 2.9, t = 2.427, df = 544, p > 0.016) but did not differ significantly in diagnostic distribution or general symptom severity as measured by the PANSS and GAF. There were no significant differences in age, educational level, or general clinical characteristics between immigrants that completed the questionnaire and those who did not. Of those who completed the questionnaire, however, there were significantly more immigrants from Europe (67.7% vs. 49.7%, *x*^2 ^= 5.045, df = 1, p < 0.025), and significantly fewer FGIs (49.2% vs. 71.4%, *x*^2 ^= 7.430, df = 1, p < 0.006) and Asian immigrants (52.6% vs. 75.5%, *x*^2 ^= 7.447, df = 1, p < 0.006) than in the total immigrant TOP-sample. We did not find any significant differences in immigrant origins, generation, or diagnosis between participants recruited from the inpatient or outpatient facilities.

### Statistical Analysis

Statistical analysis was performed using PASW Statistics 18 (SPSS inc., Chicago). The level of significance was preset to p < 0.05 (two tailed). Internal consistency of the scale measuring perceived discrimination was analyzed with Cronbach's α reliability test. Group differences were investigated with Student's t-tests (continuous variables) and chi-square tests (categorical variables). European, Asian, and African immigrants constituted the largest immigrant groups in this sample, and differences between these three groups were compared using analysis of variance (ANOVA) with Bonferroni post-hoc comparisons.

Student's t-tests for categorical variables and Pearson's correlations for continuous variables were used to explore the bivariate relationship between symptoms (PANSS positive and depression/anxiety) and demographic variables (age, sex, years of education, employment or student status, immigrant generation, and geographic origins), diagnostic variables (principle diagnosis, substance abuse/addiction diagnosis), and assessment of perceived discrimination and denial of resources.

Mediation was explored using the model proposed by Baron and Kenny [42]. We conducted simple linear regression analysis of the relationships between geographical origin, perceived discrimination, and positive and depression/anxiety symptoms, and analyzed mediation with the two-block multiple regression of relationships found to be significant in the previous analysis.

Multiple hierarchical regression analysis was conducted to assess relationships between positive and depression/anxiety symptoms and perceived discrimination/denial of resources, adjusting for significant or hypothesis-driven confounders. Models contained the variable diagnosis (block 1), immigrant generation and geographical origins (block 2), and perceived discrimination and denial of resources (block 3). Due to differences in the patterns of significant associations with symptoms, occupational status (employed, student, or unemployed) was included in block 1 of the analysis of depression/anxiety symptoms, while years of education was included in the analysis of positive symptoms.

## Results

In our sample of 90 immigrants, 24 (26.7%) were from Europe, 19 (21.1%) from Africa, 42 (46.7%) from Asia including Turkey, two (2.2%) from North America, and 3 (3.3%) from South America. A total of 59 were FGIs (66%). The FGIs were significantly older than the SGIs, were more often married, and had lower GAF-f scores of global functioning (Table 1). Immigrants from the European continent included in the study were more often female. They also had a higher incidence of bipolar disorder than immigrants from Africa.

**Table 1:** Comparison of demographic and clinical characteristics between immigrant generations and geographical origins

Continious variables Mean ± sd	1 gen (N = 59)	2 Gen (N = 31)	t-test (df = 88)	African (N = 19)	Asian (N = 42)		European (N = 24)	**F**^**2/82**^
Age (mean years)	32.95 ± 10.1	24.84 ± 5.9	4.120**	31.11 ± 11.1	29.76 ±9.0		29.33 ± 9.6	
Education (mean years)	12.68 ± 3.9	11.97 ± 2.8		11.18 ±2.8	11.90 ±3.5		13.48 ± 3.7	
GAF - symptom	43.64 ± 10.1	45.13 ± 12.5		40.16 ±6.1	43.29 ±11.7		47.08 ± 11.4	
GAF - function	42.2 ± 8.9	47.06 ± 11.5	-2.227*	41.68 ±8.9	42.17 ±10.2		47.46 ± 9.7	

**Categorical variables N (%)**			**χ^2 ^(df = 1)**					

Male	32 (54.2)	18 (58.1)		16 (84.2)	25 (59.5)	>^A^	7 (29.2)	7.664**
Married/co-inhabitant	24 (40.7)	5 (16.1)	5.608*	7 (36.8)	15 (35.7)		5 (20.8)	
Employed/Student	17 (28.8)	8 (25.8)		4 (21.1)	9 (21.4)		9 (37.5)	
Schizophrenia spectrum	29 (49.2)	15 (48.4)		12 (63.2)	22 (52.4)		9 (37.5)	
Bipolar disorder	17 (28.8)	6 (19.4)		1 (5.3)	10 (23.8)	<^B^	10 (41.7)	4.020*
Major depression/Other	13 (22)	10 (32.3)		6 (31.6)	10 (23.8)		5 (20.8)	

*p < .05, ** p < .001, ^A ^Post-hoc Bonferroni shows significant variance between immigrants from Europe and both Asia/Africa at 0.05 level,

^B ^Post-hoc Bonferroni shows significant variance between immigrants from Europe and Africa only at 0.05 level.

Schizophrenia spectrum includes DSM-IV diagnoses schizophrenia, schizoaffective- and schizophreniform disorder.

Perceived discrimination was significantly associated with PANSS positive (r = 0.26, p < 0.05) and depression/anxiety symptoms (r = 0.28, p < 0.01), but not negative (r = -.05, p = 0.614), cognitive (r = 0.04, p = 0.691) or excitement symptoms (r = 0.16, p = 0.122). Similarly, denial of resources was associated with more severe positive and depression/anxiety symptoms. Bivariate correlations between relevant variables and positive and depression/anxiety symptom severity are shown in Table 2.

**Table 2:** Bivariate analysis of discrimination measures and possible confounders with PANSS positive and depression/anxiety symptoms

	N	Positive symptoms				Depression/anxiety symptoms			
Variables	yes/no	Yes	No	**t-test**^**df88**^	*r*	Yes	No	**t-test**^**df88**^	*r*
Schizophrenia spectrum	44/46	11.89 ± 4.4	8.57 ± 3.9	-3.813**	.377**	17.82 ± 5.2	15.93 ± 5.4	ns	.177
Bipolar disorder	23/67	6.78 ± 3.8	11.36 ± 4.0	4.762**	-.453**	14.83 ± 5.2	17.55 ± 5.2	2.157*	-.224*
Major depression/other psychosis	23/67	10.35 ± 3.2	10.13 ± 4.8	ns	.021	17.04 ± 5.4	16.79 ± 5.4	ns	.021
Substance abuse/dependency	24/66	11.33 ± 4.3	9.77 ± 4.5	ns	.157	18.08 ± 5.7	16.41 ± 5.2	ns	.140
European	24/66	7.63 ± 3.5	11.12 ± 4.4	3.512**	-.351**	14.92 ± 3.9	17.56 ± 5.6	2.119*	-.220*
Asian including Turkish	42/48	10.81 ± 4.4	9.65 ± 10.8	ns^A^	.132	17.33 ± 5.8	16.44 ± 5.0	ns	.084
African	19/71	12.63 ± 3.8	9.54 ± 4.4	-2.806*	.287*	19.05 ± 5.3	16.27 ± 5.2	-2.057*	.214*
First generation immigrants	59/31	10.42 ± 4.6	9.74 ± 4.1	ns	-.073	17.49 ± 5.1	15.65 ± 5.7	ns	-.165
Male	50/40	10.92 ± 4.2	9.28 ± 4.6	ns	-.185	17.18 ± 5.4	16.45 ± 5.3	ns	-.068
Employed/Student	25/65	8.4 ± 3.5	10.88 ± 4.6	2.439*	-.252*	14.32 ± 3.9	17.83 ± 5.5	2.911**	-.296**
Age					.053				.123
Education					-.309**				-.177
**Perceived discrimination**					.264*				.282*
**Denial of resources**	35/54	12 ± 4.5	9.13 ± 4.0	-3.148**	.320**	18.34 ± 5.1	15.87 ± 5.4	-2.168*	.226*

For categorical variables means ± SD are presented, * p < .05, ** p < .005,

^A ^One-way ANOVA of symptom variation between European, African and Asian immigrants using post-hoc Bonferroni shows significant variance between Asian and European immigrants at 0.01 level (F = 8.770^2/82^, p < .001)

Schizophrenia spectrum includes DSM-IV diagnoses schizophrenia, schizoaffective- and schizophreniform disorder.

African immigrants had the most severe positive and depression/anxiety symptoms, and reported significantly higher perceived discrimination (t = 2.472, df = 88, p < 0.015). Asian immigrants had significantly higher positive symptoms than European immigrants. The least severe symptoms were found among immigrant from Europe, participants with bipolar disorder, and the employed.

Multiple linear regression analyses (Table 3) revealed that the association between African immigrant status and symptom severity was reduced when perceived discrimination was added to the analysis. These results demonstrated that positive and depression/anxiety symptoms were partially mediated by perceived discrimination for African immigrants in this model.

**Table 3:** Mediation effect of perceived discrimination on association between African immigrants and positive and depression/anxiety symptoms

	Model 1 *B coefficient (se)*	*P <*	Model 2 *B coefficient (se)*	*P <*
	**Positive symptoms**			

Perceived discrimination			1.217 (.620)	.053
Geographical origins				
Africa vs. All other	3.096 (1.103)	.006	2.535 (1.123)	.027

	**Depression/anxiety symptoms**			

Perceived discrimination			1.749 (.754)	.023
Geographical origins				
Africa vs. All other	2.785 (1.354)	.043	1.978 (1.366)	.151

Model 1 shows a simple linear regression analysis between African immigrants and symptoms.

Model 2 shows multiple regression analyses between African immigrants and symptoms, including perceived discrimination as a mediating variable.

Expanding the multiple hierarchical regression analysis revealed that perceived discrimination and denial of resources were still significantly associated with PANSS positive symptoms even after controlling for other relevant potentially confounding factors (Table 4). The full model explained 34% of the variance, with the discrimination measures alone explaining 11%. The same analysis using occupational status instead of educational level showed that perceived discrimination retained a significant association with PANSS depression/anxiety symptoms (Table 5) after controlling for relevant confounders. In this case, the model explained 21% of the variance, with the discrimination measures contributing 9.5%. Generational status (FGI or SGI) did not contribute significantly to any of these models.

**Table 4:** Multiple hierarchical regression between discrimination measures and PANSS positive symptoms including possible confounders

Block no., Variables	**R**^**2 **^**change**	Beta (SE)	95% CI for B	t-test	p-value
Constant		9.171 (2.456)	4.285 - 14.057	3.734	.000
1					
Education (years)	.208**	-.179 (.133)	-.442 - .085	-1.347	.182
Bipolar disorder		-3.936 (1.047)	-6.017 - -1.855	-3.760	.000
2					
Generation (1 First, 2 Second)	.073*	-1.059 (.858)	-2.765 - .648	-1.233	.221
European origin		-2.411 (.950)	-4.300 - -.522	-2.538	.013
3					
Perceived discrimination	.107**	1.148 (.547)	.059 - 2.236	2.097	.039
Denial of resources		2.025 (.822)	.390 - 3.659	2.464	.016

Final model, ΔR^2 ^= .344, F^6/82 ^= 8.680, p < .001

** p <. 001, * p < .05

**Table 5:** Multiple hierarchical regression between discrimination measures and PANSS depression/anxiety symptoms including possible confounders

Block no., Variables	**R**^**2 **^**change**	Beta (SE)	95% CI for B	t-test	p-value
Constant		15.677 (2.528)	10.647 - 20.706	6.200	.000
1					
Employed/Student	.112*	-2.970 (1.256)	-5.467 - -.473	-2.365	.020
Bipolar disorder		-2.006 (1.309)	-4.608 - .596	-1.533	.129
2					
Generation (1 First, 2 Second)	.058	-1.999 (1.121)	-4.227 - .230	-1.784	.078
European origin		-1.878 (1.245)	-4.353 - .597	-1.509	.135
3					
Perceived discrimination	.095*	1.929 (.709)	.519 - 3.339	2.721	.008
Denial of resources		1.125 (1.089)	-1.042 - 3.292	1.033	.305

Final model, ΔR^2 ^= .211, F^6/82 ^= 4.931, p < .001, * p < .01

## Discussion

Our main finding was that perceived discrimination was associated with more severe positive and depression/affective symptoms among immigrants with psychosis. In contrast, perceived discrimination was not significantly associated with the severity of negative, cognitive, or excitement symptoms. Perceived discrimination had a partial mediating effect on the severity of positive and depression/anxiety symptoms in African immigrants. Perceived discrimination also has a strong independent effect on the severity of positive and depression/anxiety symptoms even after controlling for diagnostic group, immigrant generation, and geographic origins.

Our results are in accord with earlier findings demonstrating an association between discrimination and delusional ideation (a positive symptom) [16,26]. Furthermore, a recent meta-analysis found that discrimination, independent of ethnicity, was related to poor mental health, including a higher incidence of depressive symptoms [12]. This same meta-analysis also found a clear relationship between discrimination and measures of physical stress, such as elevated blood pressure, heart rate, and cortisol secretion. This may partly explain the association between perceived discrimination and somatic concerns, anxiety, and tension that were all sub-items of the depression/anxiety factor used in our study. A recent study of a large sample of Puerto Ricans in the USA concluded that depressive symptoms were a mediator of the effect of perceived discrimination on a number of somatic conditions [43]. We have previously shown that immigrants who have migrated from the Southern to the Northern Hemispheres and patients with psychotic disorders in general are more prone to vitamin D deficiency, another factor which is associated with depressive symptoms [44]. Including levels of vitamin D might have enhanced the predictive value of our model, but unfortunately we did not have access to vitamin D measures in all participating patients.

We found that immigrants from outside Europe had more severe symptoms than immigrants from Europe. Early research from the beginning of the 19th century reported increased rates of schizophrenia among immigrants from Britain and Continental Europe to Canada, and among Norwegian immigrants to the USA [45,46]. Seeman [6] suggested that these immigrant groups, although not visible minorities, did stand out in their new country because of language difficulties, higher unemployment, and a history of deprivation. Perception of discrimination may engender feelings of alienation among visible minorities that in turn exacerbate symptoms. Immigrants from Europe may better integrate with the majority (Caucasian) culture, while both FGIs and SGIs from Africa and Asia are more visible and must adapt to greater cultural barriers [47]. In fact, we found that perceived discrimination was a mediator for the influence of African immigrant status on the severity of positive and depression/anxiety symptoms. These findings are of particular importance considering that the highest relative risk of developing psychotic disorders in immigrant groups was found among those migrants from areas where the majority of the population is black [1].

Based on these results, we suggest that discrimination can be an important environmental stressor leading to the development and escalation of both depression/anxiety and positive psychotic symptoms in patients with psychotic disorders, and may help explain the distinct psychopathology profiles reported in different ethnic minorities. The experience of deprivation of resources and rewards based on visible minority status may lead to feelings of hopelessness and an external locus of control, both of which are psychological mechanisms associated with depression [48]. Visible minority status may also enhance alienation and in some cases lead to actual persecution. Cultural differences can result in miscommunication between the minority and majority populations. For individuals predisposed to psychosis, these experiences can lead to enhanced suspiciousness and to psychotic episodes. This conclusion is supported by findings demonstrating that peer victimization in childhood increased the risk for psychotic symptoms, independent of prior psychopathology, family adversity, or IQ [49], and supports the hypothesis that experiences of social defeat are important in the etiology of schizophrenia [13].

It is possible that individuals who are prone to psychosis or suffering from paranoid ideation are likely to perceive neutral or ambiguous situations as discriminatory. As our study was cross-sectional, we were unable to assess the direction of the association between perceived discrimination and symptom profiles. However, a meta-analysis of 110 studies found that perceived discrimination was significantly related to negative mental health outcomes and that 12 experimental studies assessing causality found that perceived discrimination can indeed cause an increase in both physical and psychological stress responses in healthy populations, strongly supporting the causative role of discrimination [12]. Longitudinal and controlled experimental studies are needed to assess the direction of associations between perceived discrimination and symptom severity in immigrants with psychosis.

### Strengths and Limitations

Our study included a well-documented clinical sample of patients with psychotic disorders. Patients were recruited from a public health care system providing equal treatment services to all groups with extensive experience in treating patients from different cultures. The organization of the Norwegian public health care system thus ensures more representative recruitment than more socioeconomically segregated systems. Our final sample also mirrored the true demographics of the Oslo immigrant population, with the exception of a higher proportion of SGIs (and fewer FGIs). This could be a consequence of the language exclusion criterion, where we required patients to have adequate Scandinavian language skills. It is expected that more SGIs are competent in Norwegian, but this may have excluded FGI patients with poor language skills.

An important consideration in cross-cultural studies of psychopathology is the validity of the assessment tools. The assessment personnel in our group were trained to use the SCID-I for diagnostic purposes by watching training videos that including patients from different ethnic and cultural backgrounds. The instrument used to assess symptom severity (PANSS) was originally developed in an inter-ethnic population, thus strengthening its cultural validity. Diagnostic evaluations and symptom assessments were based on face to face interviews rather than patient journals, databases, or surveys. However, it is unavoidable that the assessor is aware of each patient's ethnicity, and this could influence diagnosis. In addition, the ethnic sub-groups were small, possibly limiting the generalization of our findings. The cross-sectional design of this study prevents us from making causal inferences, and we cannot make any inferences of risk.

## Conclusions

We have shown that perceived discrimination among immigrants with psychosis is associated with more severe positive and depression/anxiety symptoms, and that these perceptions function as a mediator of illness severity for immigrants from Africa. We suggest that stressful environmental factors lead to heightened risk for psychosis and influence the specific symptom profile and severity. In a world with ever increasing migration and cross-cultural interactions, this result has important implications for both the prevention and treatment of minorities suffering from psychotic illnesses. Future studies should focus on the possible association between context-specific stressors and symptoms in other immigrant populations.

## Competing interests

The authors declare that they have no competing interests.

## Authors' contributions

AB conceived of the study, collected data, performed and interpreted the statistical analysis and drafted the manuscript, IM conceived and administrated the study, interpreted statistical results, edited and revised the manuscript, JIR performed and interpreted statistical analysis, edited and revised the manuscript, KLR acquired data, contributed to drafting the manuscript, and edited and revised the manuscript, SL acquired data and edited and revised the manuscript, TVL acquired data, contributed to drafting the manuscript, and edited and revised the manuscript, OAA conceived and administered the study, contributed to drafting the manuscript, and edited and revised the manuscript, EH participated in conception of the study, interpretation of results, and edited and revised the manuscript. All authors read and approved the final manuscript.

## Pre-publication history

The pre-publication history for this paper can be accessed here:

http://www.biomedcentral.com/1471-244X/11/77/prepub

## References

[B1] Cantor-Graae E Selten J Schizophrenia and Migration: A Meta-Analysis and Review Am J Psychiatry 2005 162 12 24 10.1176/appi.ajp.162.1.12 15625195

[B2] Coid JW Kirkbride JB Barker D Cowden F Stamps R Yang M Jones PB Raised Incidence Rates of All Psychoses Among Migrant Groups: Findings From the East London First Episode Psychosis Study Arch Gen Psychiatry 2008 65 1250 1258 10.1001/archpsyc.65.11.1250 18981336

[B3] Weiser M Werbeloff N Vishna T Yoffe R Lubin G Shmushkevitch M Davidson M Elaboration on immigration and risk for schizophrenia Psychol Med 2008 38 1113 1119 17988415 10.1017/S003329170700205X

[B4] Bourque F van der Ven E Malla A A meta-analysis of the risk for psychotic disorders among first- and second-generation immigrants Psychol Med 2010 1 14 10.1017/S0033291710001406 20663257

[B5] Morgan C Hutchinson G The social determinants of psychosis in migrant and ethnic minority populations: a public health tragedy Psychol Med 2010 40 705 709 10.1017/S0033291709005546 19335938

[B6] Seeman MV Canada: Psychosis in the Immigrant Caribbean Population Int J Soc Psychiatry 2010 Published online ahead of print April 13, 2010 10.1177/0020764010365979 20388719

[B7] Collip D Myen-Germeys I Van Os J Does the concept of "sensitization" provide a plausible mechanism for the putative link between the environment and schizophrenia? Schizophr Bull 2008 34 220 225 18203757 10.1093/schbul/sbm163 PMC2632409

[B8] Fossion P Servais L Rejas MC Ledoux Y Pelc I Minner P Psychosis, migration and social environment: an age--and--gender controlled study Eur Psychiatry 2004 19 338 343 10.1016/j.eurpsy.2004.04.010 15363471

[B9] Karlsen S Nazroo JY McKenzie K Bhui K Weich S Racism, psychosis and common mental disorder among ethnic minority groups in England Psychol Med 2005 35 1795 1803 10.1017/S0033291705005830 16194282

[B10] Veling W Selten JP Susser E Laan W Mackenbach JP Hoek HW Discrimination and the incidence of psychotic disorders among ethnic minorities in The Netherlands Int J Epidemiol 2007 36 761 768 10.1093/ije/dym085 17517810

[B11] Eaton W Harrison G Ethnic disadvantage and schizophrenia Acta Psychiatr Scand 2000 102 38 43 10.1034/j.1600-0447.2000.00007.x 11261638

[B12] Pascoe EA Smart Richman L Perceived discrimination and health: a meta-analytic review Psychol Bull 2009 135 531 554 19586161 10.1037/a0016059 PMC2747726

[B13] Selten JP Cantor-Graae E Social defeat: risk factor for schizophrenia? Br J Psychiatry 2005 187 101 102 10.1192/bjp.187.2.101 16055818

[B14] Fuchs T Life events in late paraphrenia and depression Psychopathology 1999 32 60 69 10.1159/000029069 10026450

[B15] Janssen I Hanssen M Bak M Biji R Vollebergh W McKenzie K van Os J Evidence that ethnic group effects on psychosis risk are confounded by experience of discrimination [Abstract] Eur Psychiatry 2002 17 83 84

[B16] Janssen I Hanssen M Bak M Bijl RV De Graaf R Vollebergh W McKenzie K van Os J Discrimination and delusional ideation Br J Psychiatry 2003 182 71 76 10.1192/bjp.182.1.71 12509322

[B17] Adebimpe VR Klein HE Fried J Hallucinations and delusions in black psychiatric patients J Natl Med Assoc 1981 73 517 520 7241610 PMC2552720

[B18] Adebimpe VR Chu CC Klein HE Lange MH Racial and geographic differences in the psychopathology of schizophrenia Am J Psychiatry 1982 139 888 891 7091405 10.1176/ajp.139.7.888

[B19] Mukherjee S Shukla S Woodle J Rosen AM Olarte S Misdiagnosis of schizophrenia in bipolar patients: a multiethnic comparison Am J Psychiatry 1983 140 1571 1574 6650685 10.1176/ajp.140.12.1571

[B20] Harvey I Williams M McGuffin P Toone BK The functional psychoses in Afro-Caribbeans Br J Psychiatry 1990 157 515 522 10.1192/bjp.157.4.515 2131132

[B21] Strakowski SM McElroy SL Keck PE West SA Racial influence on diagnosis in psychotic mania J Affect Disord 1996 39 157 162 10.1016/0165-0327(96)00028-6 8827426

[B22] Barrio C Yamada AM Atuel H Hough RL Yee S Berthot B Russo PA A tri-ethnic examination of symptom expression on the positive and negative syndrome scale in schizophrenia spectrum disorders Schizophr Res 2003 60 259 269 12591588 10.1016/s0920-9964(02)00223-2

[B23] Kennedy N Boydell J van Os J Murray RM Ethnic differences in first clinical presentation of bipolar disorder: results from an epidemiological study J Affect Disord 2004 83 161 168 10.1016/j.jad.2004.06.006 15555709

[B24] Veling W Selten JP Mackenbach JP Hoek HW Symptoms at first contact for psychotic disorder: Comparison between native Dutch and ethnic minorities Schizophr Res 2007 95 30 38 10.1016/j.schres.2007.06.024 17669627

[B25] Vanheusden K Mulder CL van der Ende J Selten JP van Lenthe FJ Verhulst FC Mackenbach JP Associations between ethnicity and self-reported hallucinations in a population sample of young adults in The Netherlands Psychol Med 2008 38 1095 1102 18070372 10.1017/S0033291707002401

[B26] Bentall RP Corcoran R Howard R Blackwood N Konderman P Persecutory Delusions: A Review and Theoretical Intergration Clin Psychol Rev 2001 21 1143 1192 10.1016/S0272-7358(01)00106-4 11702511

[B27] Haasen C Yagdiran O Mass R Krausz M Schizophrenic disorders among Turkish migrants in Germany. A controlled clinical study Psychopathology 2001 34 203 208 10.1159/000049308 11549931

[B28] Klonoff EA Landrine H Is skin color a marker for racial discrimination? Explaining the skin color-hypertension relationship J Behav Med 2000 23 329 338 10.1023/A:1005580300128 10984862

[B29] Cooper C Morgan C Byrne M Dazzan P Morgan K Hutchinson G Doody GA Harrison G Leff J Jones P Ismael K Murray R Bebbington P Fearon P Perceptions of disadvantage, ethnicity and psychosis Br J Psychiatry 2008 192 185 190 10.1192/bjp.bp.107.042291 18310577

[B30] World Medical Association Declaration of Helsinki: Ethical Principles for Medical Research Involving Human Subjects http://www.wma.net/en/30publications/10policies/b3/17c.pdf 10.1191/0969733002ne486xx 16010903

[B31] American Psychiatric Association Diagnostic and statistical manual of mental disorders: DSM-IV 1994 4 Washington, D.C.: American Psychiatric Association

[B32] Ventura J Liberman RP Green MF Shaner A Mintz J Training and quality assurance with the Structured Clinical Interview for DSM-IV (SCID-I/P) Psychiatry Res 1998 79 163 173 10.1016/S0165-1781(98)00038-9 9705054

[B33] Kay SR Fiszbein A Opler LA The positive and negative syndrome scale (PANSS) for schizophrenia Schizophr Bull 1987 13 261 276 3616518 10.1093/schbul/13.2.261

[B34] Lindenmeyer JP Bossie CA Kujawa M Zhu Y Canuso CM Dimensions of psychosis in patients with bipolar mania as measured by the positive and negative syndrome scale Psychopathology 2008 41 264 270 10.1159/000128325 18441528

[B35] Fresan A De la Fuente-Sandoval C Loyzaga C Garcia-Anaya M Meyenberg N Nicolini H Apiquian R A forced five-dimensional factor analysis and concurrent validity of the Positive and Negative Syndrome Scale in Mexican schizophrenic patients Schizophr Res 2005 72 123 129 10.1016/j.schres.2004.03.021 15560957

[B36] Pedersen G Hagtvet KA Karterud S Generalizability studies of the Global Assessment of Functioning-Split version Compr Psychiatry 2007 48 88 94 10.1016/j.comppsych.2006.03.008 17145287

[B37] Berry JW Phinney JS Sam DL Vedder P Immigrant Youth in Cultural Transition; Acculturation, identity, and Adaptation Across National Contexts 2006 1 New Jersey: Lawrence Erlbaum Associates, Inc21548949

[B38] Oslo Health Study (HUBRO and youth part) http://www.fhi.no/eway/default.aspx?pid=233&trg=MainArea_5661&MainArea_5661=5631:0:15,4385:1:0:0:::0:0

[B39] Veling W Hoek HW Mackenbach JP Perceived discrimination and the risk of schizophrenia in ethnic minorities: a case-control study Soc Psychiatry Psychiatr Epidemiol 2008 43 953 959 10.1007/s00127-008-0381-6 18575790

[B40] The Oslo Immigrant Health Study, Norway http://www.fhi.no/dav/906123CAA9.pdf

[B41] Daugstad G Ed. Immigration and Immigrants, 2008 2008 Kongsvinger: Statistics Norway

[B42] Baron RM Kenny DA The moderator-mediator variable distinction in social psychological research: conceptual, strategic, and statistical considerations J Pers Soc Psychol 1986 51 1173 1182 3806354 10.1037//0022-3514.51.6.1173

[B43] Todorova IL Falcon LM Lincoln AK Price LL Perceived discrimination, psychological distress and health Sociol Health Illn 2010 32 843 861 10.1111/j.1467-9566.2010.01257.x 20649891 PMC4437189

[B44] Berg AO Melle I Torjesen PA Lien L Hauff E Andreassen OA A cross-sectional study of vitamin D deficiency among immigrants and Norwegians with psychosis compared to the general population J Clin Psychiatry 2010 71 1598 1604 10.4088/JCP.09m05299yel 20441728

[B45] Ödegaard Ö Emigration and insanity Acta Psychiatrica Et Neurologica Scandinavica 1932 Suppl 4 1 206

[B46] Smith GN Boydell J Murray RM Flynn S McKay K Sherwood M Honer WG The incidence of schizophrenia in European immigrants to Canada Schizophr Res 2006 87 205 211 10.1016/j.schres.2006.06.024 16905294

[B47] Wiley S Perkins K Deaux K Through the looking glass: Ethnic and generational patterns of immigrant identity Int J Intercult Rel 2008 32 385 398 10.1016/j.ijintrel.2008.04.002

[B48] Benassi VA Sweeney PD Dufour CL Is there a relation between locus of control orientation and depression? J Abnorm Psychol 1988 97 357 367 3057032 10.1037//0021-843x.97.3.357

[B49] Schreier A Wolke D Thomas K Horwood J Hollis C Gunnell D Lewis G Thompson A Zammit S Duffy L Salvi G Harrison G Prospective study of peer victimization in childhood and psychotic symptoms in a nonclinical population at age 12 years Arch Gen Psychiatry 2009 66 527 536 10.1001/archgenpsychiatry.2009.23 19414712

